# Characteristics of trauma patients with creatine kinase elevation

**DOI:** 10.1186/cc14362

**Published:** 2015-03-16

**Authors:** N Assanangkornchai, O Akaraborworn, C Kongkamol, K Kaewsaengrueang

**Affiliations:** 1 Prince of Songkla University, Hatyai, Thailand

## Introduction

Rhabdomyolysis is a condition that results in the release of mainly creatine kinase (CK) and myoglobin from the breakdown of myocytes. Myoglobin has been known to cause renal failure (RF) and the CK level is routinely used as an indicator. A CK level >5,000 U/l was found to be associated with the risk of RF [1]. However, data are lacking on the level of CK to predict RF, especially in general trauma patients. The purpose of this study was to determine the initial CK level that predicts markedly elevated CK and the characteristics of trauma patients with elevated CK.

## Methods

Data from the Songklanagarind Hospital trauma registry were reviewed over 1 year (January 2013 to December 2013). Patients with at least two records of CK and creatinine (Cr) levels were included. Creatine kinase levels were analyzed during the first 3 days of hospital admission. RF was defined as a Cr increment >0.3 mg/dl within 48 hours.

## Results

Of the 1,491 patients admitted to the trauma service, 47 patients had CK levels drawn twice. These patients had a mean age of 32 years and a median Injury Severity Score (ISS) of 14. The predominant mechanism of injury was motorcycle crash. Only three patients developed RF. The median CK during the first 3 days after admission was 3,088 (IQR 1,327, 6,072) U/l. The CK peaked at 11 hours after admission at a mean value of 16,114.167 (SD 34,010.80) U/l. There were no significant differences in demographic data, ISS scores and fluid balance between the groups of CK level over or below 5,000 U/l. A mean positive fluid balance observed; however, initial CK was significantly different between the two groups. None of the patients with initial CK of <900 U/l had a peak of CK >5,000 U/l.

## Conclusion

Trauma patients had varying levels of elevated CK. Initial CK shows a promising result as a predictor of high peak CK levels. A larger sample size is needed to demonstrate the predictors of RF in trauma patients with elevated CK levels.
